# Feedback Regulation of Syk by Protein Kinase C in Human Platelets

**DOI:** 10.3390/ijms21010176

**Published:** 2019-12-25

**Authors:** Stephanie Makhoul, Stephanie Dorschel, Stepan Gambaryan, Ulrich Walter, Kerstin Jurk

**Affiliations:** 1 Center for Thrombosis and Hemostasis (CTH), University Medical Center Mainz of the Johannes Gutenberg University Mainz, 55131 Mainz, Germany; stephanie.makhoul@uni-mainz.de (S.M.); stephanie.dorschel@gmail.com (S.D.); s.gambaryan@klin-biochem.uni-wuerzburg.de (S.G.); ulrich.walter@uni-mainz.de (U.W.); 2 Sechenov Institute of Evolutionary Physiology and Biochemistry, Russian Academy of Sciences, 194223 St. Petersburg, Russia

**Keywords:** spleen tyrosine kinase (Syk), protein kinase C, cyclic adenosine monophosphate (cAMP), platelets, glycoprotein VI, glycoprotein Ibα

## Abstract

The spleen tyrosine kinase (Syk) is essential for immunoreceptor tyrosine-based activation motif (ITAM)-dependent platelet activation, and it is stimulated by Src-family kinase (SFK)-/Syk-mediated phosphorylation of Y352 (interdomain-B) and Y525/526 (kinase domain). Additional sites for Syk phosphorylation and protein interactions are known but remain elusive. Since Syk S297 phosphorylation (interdomain-B) was detected in platelets, we hypothesized that this phosphorylation site regulates Syk activity via protein kinase C (PKC)-and cyclic adenosine monophosphate (cAMP)-dependent pathways. ADP, the GPVI-agonist convulxin, and the GPIbα-agonist echicetin beads (EB) were used to stimulate human platelets with/without effectors. Platelet aggregation and intracellular messengers were analyzed, along with phosphoproteins, by immunoblotting using phosphosite-specific antibodies or phos-tags. ADP, convulxin, and EB upregulated Syk S297 phosphorylation, which was inhibited by iloprost (cAMP pathway). Convulxin-stimulated Syk S297 phosphorylation was stoichiometric, transient, abolished by the PKC inhibitor GF109203X, and mimicked by the PKC activator PDBu. Convulxin/EB stimulated Syk S297, Y352, and Y525/526 phosphorylation, which was inhibited by SFK and Syk inhibitors. GFX and iloprost inhibited convulxin/EB-induced Syk S297 phosphorylation but enhanced Syk tyrosine (Y352/Y525/526) and substrate (linker adaptor for T cells (LAT), phospholipase γ2 (PLC γ2)) phosphorylation. GFX enhanced convulxin/EB-increases of inositol monophosphate/Ca^2+^. ITAM-activated Syk stimulates PKC-dependent Syk S297 phosphorylation, which is reduced by SFK/Syk/PKC inhibition and cAMP. Inhibition of Syk S297 phosphorylation coincides with enhanced Syk activation, suggesting that S297 phosphorylation represents a mechanism for feedback inhibition in human platelets.

## 1. Introduction

Human platelets are small circulating blood cells, which control, monitor, and preserve the integrity of the vessel wall. These anucleate cells prevent blood loss during vascular injury and, on the other hand, have prominent roles in thrombotic, inflammatory, and immune pathologies [1,2]. After vascular injury, distinct platelet receptors sense and bind newly exposed proteins immobilized within the extracellular matrix or on activated endothelial cells. This is followed by receptor-mediated platelet activation resulting in a plethora of cellular responses, including cytoskeletal remodeling, integrin activation (e.g., integrin α_IIb_β_3_), degranulation, synthesis/release of thromboxane A2 (TxA2), and surface exposure of anionic phospholipids, leading to platelet shape change, adhesiveness, aggregation, and coagulant activity to form a protective thrombus [2,3]. These essential physiological processes are often impaired in diseases and are tightly controlled by numerous hormones, vasoactive factors, and adhesive proteins, which activate, modulate, or inhibit these platelet functions. Two major classes of platelet activators include soluble agonists and adhesion molecules, e.g., von Willebrand factor (vWF), collagen, fibrin, and podoplanin, which stimulate specific G-protein-coupled receptors (GPCRs) [4,5] and cell membrane-spanning adhesion receptors, respectively [6,7,8]. GPCR-coupled agonists activate phospholipase (PLC) β, elevate cytosolic Ca^2+^ concentration, and activate Ca^2+^-dependent protein kinases, such as protein kinase C (PKC), thereby inducing platelet activation [4,5].

The adhesion molecules collagen, vWF, podoplanin, fibrinogen, and fibrin bind to and activate platelet membrane receptors such as glycoprotein (GP) VI (GPVI), GPIb-V-IX, CLEC-2, integrin α_IIb_β_3_ [6,7], and subsequently Src-family tyrosine protein kinases (SFKs) causing platelet activation [8]. The SFKs tyrosine-phosphorylate other proteins/protein kinases including membrane proteins with the immunoreceptor tyrosine-based activation motif (ITAM) [6,8,9], which then recruits src homology 2 (SH2) domain-containing proteins, in particular the spleen tyrosine kinase (Syk). In human platelets, ITAM-mediated Syk activation is mediated by the Fc receptor γ-chain and the low-affinity IgG receptor FcγRIIa [10,11], while it is only mediated by the Fc receptor γ-chain in murine platelets [12,13,14,15]. Mice embryos presenting with a homozygous targeted mutation in the *Syk* gene (by deletion of one exon on *Syk* gene encoding for 41 residues in the Syk kinase domain in embryonic stem cells) die from severe hemorrhages before birth [16], and mice lacking platelet Syk were protected from arterial thrombosis and ischemic stroke [17], highlighting the important role of Syk in platelets. Tyrosine-phosphorylated ITAM proteins recruit Syk from the cytosol to the cell membrane and activate Syk via two distinct overlapping mechanisms, the described ITAM-dependent process and a tyrosine phosphorylation-dependent process [15,18,19,20]. The Syk Y-phospho-sites closely associated with activation, Y348/Y352 and Y525/Y526, are two pairs within the interdomain-B and kinase domains, respectively. Syk activation is initiated when these Y-sites are phosphorylated by SFKs or when dually Y-phosphorylated ITAM-containing membrane proteins recruit the two Syk-SH2 domains followed by Syk autophosphorylation, leading to the activation of the LAT-signalosome [18,19]. However, in addition to these Syk tyrosine phosphorylation sites involved in kinase activation, it was demonstrated, primarily with murine and human B-cells, that Syk contains multiple tyrosine, serine, and threonine phosphorylation sites, and that some of them are important for recruiting additional regulatory binding proteins [21,22,23]. Syk serine phosphorylation at S297 (S291 in murine cells) is observed in B-cells [23,24]. While Syk S291 phosphorylation in murine B-cell lines was reported to enhance Syk coupling to the B-cell antigen receptor (BCR) [24], Syk S297 phosphorylation diminished antigen–receptor signaling in human B-cell lines [23]. However, the role of Syk S297 phosphorylation in human platelets remains unknown. In our recent phosphoproteomic studies with human platelets, the cyclic adenosine monophosphate (cAMP)-elevating platelet inhibitor and stable prostacyclin analog iloprost (cAMP/protein kinase A (PKA) pathway), as well as adenosine diphosphate (ADP), affected the phosphorylation of many protein kinases including several tyrosine protein kinases such as Janus kinase (JAK) 3, activated CDC42 kinase 1(ACK1), Bruton-tyrosine kinase (BTK), and Syk [25,26]. Interestingly, ADP, which activates platelet Ca^2+^/calmodulin-dependent protein kinases such as PKC, but not iloprost, stimulated Syk S297 phosphorylation. Very recently, we established methods for the selective quantitative assessment of GPVI-and GPIbα-mediated activation and function of human platelet Syk [27,28]. We observed that cAMP-and cyclic guanosine monophosphate (cGMP)-elevating platelet inhibitors strongly inhibited GPIbα-/GPVI-mediated platelet activation but enhanced the initial Syk activation [28]. These phosphoproteomic and functional approaches suggest that there is a network of interacting protein kinases at the level of Syk in platelets [29,30].

Based on previously published data and our own findings on Syk S297 phosphorylation in human platelets, and considering the crucial Syk interdomain location of S297 [20], we hypothesized that this serine site is phosphorylated in response to the activation of several signaling pathways. In particular, we hypothesized that PKC-and cAMP-dependent pathways, via their respective protein kinases, regulate the phosphorylation of Syk S297, thereby affecting activation and/or activity of Syk in human platelets. With this approach, we aimed to show that phosphorylation of Syk S297 in platelets modulates Syk activity and, subsequently, further Syk substrates important for platelet function.

## 2. Results

### 2.1. ADP, Convulxin, and Echicetin Beads Upregulate Syk S297 Phosphorylation, Which Is Inhibited by Iloprost

**Figure 1:**
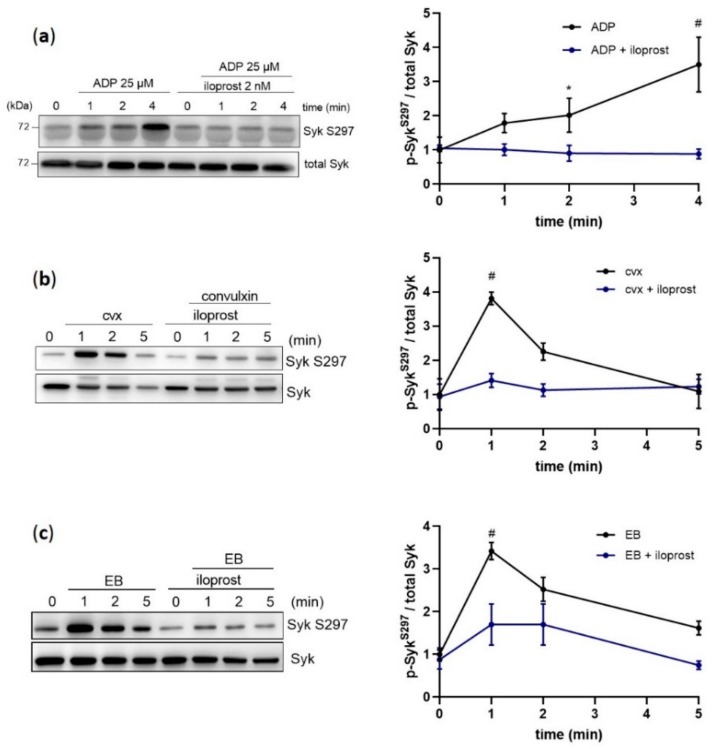
Platelet Syk S297 is upregulated by ADP, convulxin, and echicetin beads. Human washed platelets were pre-incubated with iloprost (2 nM; 3 min) at 37 °C prior to stimulation with (**a**) 25 µM ADP, (**b**) 50 ng/mL convulxin (cvx), or (**c**) 0.5% (*v*/*v*) echicetin beads (EB). Platelet aggregation was stopped after 1, 2, or 5 min of stimulation under stirring conditions by adding Laemmli buffer. Syk S297 phosphorylation was analyzed in the presence or absence of iloprost by immunoblotting compared to total protein. Quantitative data are represented as means ± SD from three independent experiments with platelets from three healthy donors. * *p* < 0.05, untreated versus iloprost-treated platelets in response to ADP at 2 min; # *p* < 0.0001, untreated versus iloprost-treated platelets at 4 min in response to ADP and at 1 min in response to cvx or EB.

Our previous phosphoproteomic studies with human platelets showed that ADP induced Syk serine phosphorylation at S297, which is located in the interdomain-B of Syk [26]. Using a phosphospecific antibody against this site, we investigated the regulation of this phosphorylation site by ADP, by its functional inhibitor (iloprost) and by agonists, which activate platelets via ITAM-/Syk-dependent mechanisms. As expected, ADP-induced platelet aggregation was completely inhibited by iloprost (Supplementary Materials Figure S1a). ADP increased Syk S297 phosphorylation 3–4-fold within 4 min of stimulation, which was strongly inhibited by iloprost (Figure 1a). Then, a rapid (within 1 min) but transient phosphorylation of S297 was observed upon platelet stimulation with the selective GPVI agonist convulxin (cvx), as well as with the specific GPIbα agonist echicetin beads (EB) (Figure 1b,c), which was also strongly inhibited by iloprost. Furthermore, cvx/EB-induced S297 phosphorylation was significantly downregulated by the blockage of the TxA2 receptor and the ADP receptor P2Y_12_ (Supplementary Materials Figure S2a,b). These data demonstrate that Syk S297 is upregulated by distinct signaling pathways in human platelets and, with both GPIbα and GPVI, is significantly dependent on the secondary mediators ADP and TxA2.

### 2.2. Transient GPVI/GPIbα-Stimulated Syk S297 Phosphorylation Parallels Syk Tyrosine Phosphorylation

**Figure 2:**
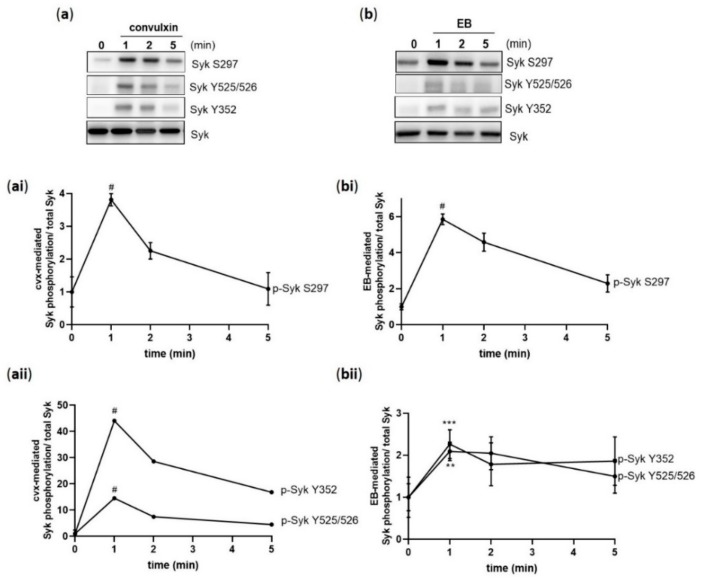
Cvx and EB induce a transient Syk S297 phosphorylation in parallel with Syk tyrosine phosphorylation. Washed human platelets were stimulated under stirring conditions with (**a**) 50 ng/mL cvx or (**b**) 0.5% (*v*/*v*) EB. (**ai**,**bi**) Syk phosphorylation on S297 and tyrosine sites (525/526 and 352) mediated by cvx or by EB were analyzed in a time-dependent manner (1, 2, and 5 min) compared to total Syk. (**aii**,**bii**) The kinetics of the phosphorylation patterns are represented as means ± SD from three independent experiments with platelets from three healthy donors. # *p* < 0.0001, *** *p* < 0.001, ** *p* < 0.01, untreated versus cvx-or EB-stimulated platelets at 1 min.

To better understand a possible link between the phosphorylation of S297 and Syk activation, we studied simultaneously the phosphorylation of Syk on S297, on Y525/526 (a Syk activation marker), and on Syk Y352 (important for Syk activity enhancement and for binding other proteins). Syk S297 showed a transient phosphorylation, which increased within 1 min of stimulation and then decreased both with convulxin (Figure 2a,ai) and EB (Figure 2b,bi) stimulation. A very similar transient time course was observed with Syk Y525/526 and Y352 phosphorylation in response to cvx (Figure 2a,aii) and EB (Figure 2b,bii). In contrast, ADP did not induce Syk Y525/526 or Y352 phosphorylation [26]. The observed Syk phosphorylation declined after initial stimulation, suggesting that a protein phosphatase is active.

### 2.3. Syk S297 Phosphorylation Is Dependent on Src Family Kinases (SFKs) and Syk Kinase Activity

**Figure 3:**
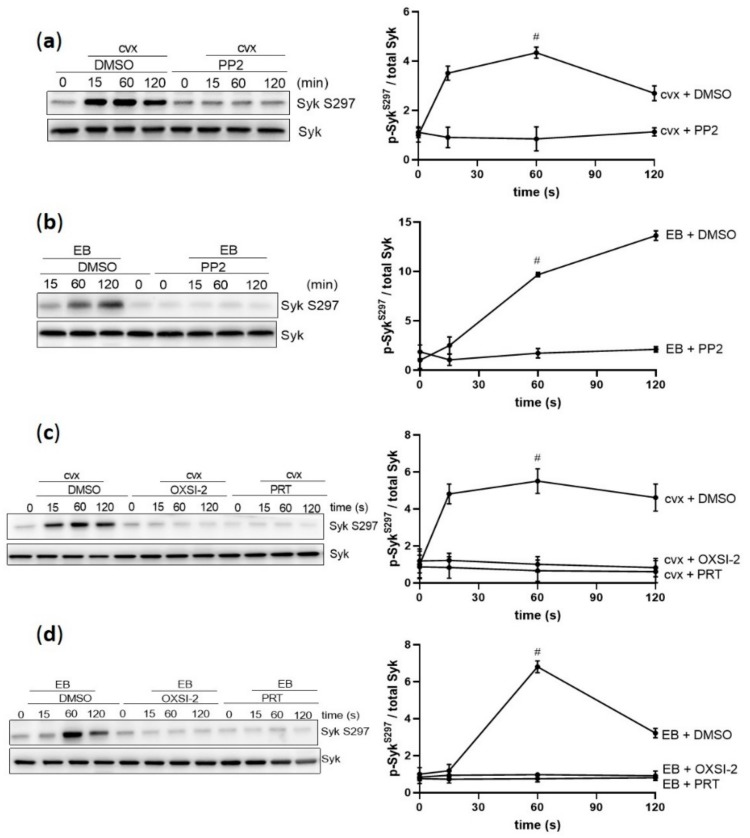
Cvx-and EB-mediated Syk S297 upregulation is dependent on SFKs and Syk activation. Washed human platelets were pre-incubated with vehicle control (DMSO) or with the SFK inhibitor PP2 (10 µM) for 5 min at 37 °C prior to stimulation, under stirring conditions with (**a**) 50 ng/mL cvx or (**b**) 0.5% (*v*/*v*) EB or with two different Syk inhibitors, OXSI-2 (2 µM) and PRT-060318 (1 µM) for 5 min at 37 °C prior to stimulation with (**c**) cvx or (**d**) EB. Phosphorylation of Syk S297 was analyzed by immunoblotting compared to total Syk. Data are represented as means ± SD from three independent experiments with platelets from three healthy donors. # *p* < 0.0001, DMSO versus inhibitor-treated (PP2, OXSI-2, or PRT-060318) platelets at 1 min in response to cvx or EB.

EB-/cvx-mediated Syk tyrosine phosphorylation is mediated by SFKs and Syk autophosphorylation [18,19,28]. Therefore, we evaluated the role of SFKs and Syk for Syk S297 phosphorylation. The SFK inhibitor PP2 abolished Syk S297 phosphorylation induced by cvx and EB (Figure 3a,b), which was also observed with the two different Syk inhibitors OXSI-2 and PRT-060318 (Figure 3c,d). These data indicate that GPVI-/GPIbα–increased Syk S297 phosphorylation is highly dependent on SFK-stimulated Syk kinase activity.

### 2.4. Convulxin-Induced Syk S297 Phosphorylation Is Stoichiometric and Abolished by Inhibition of PKC

**Figure 4:**
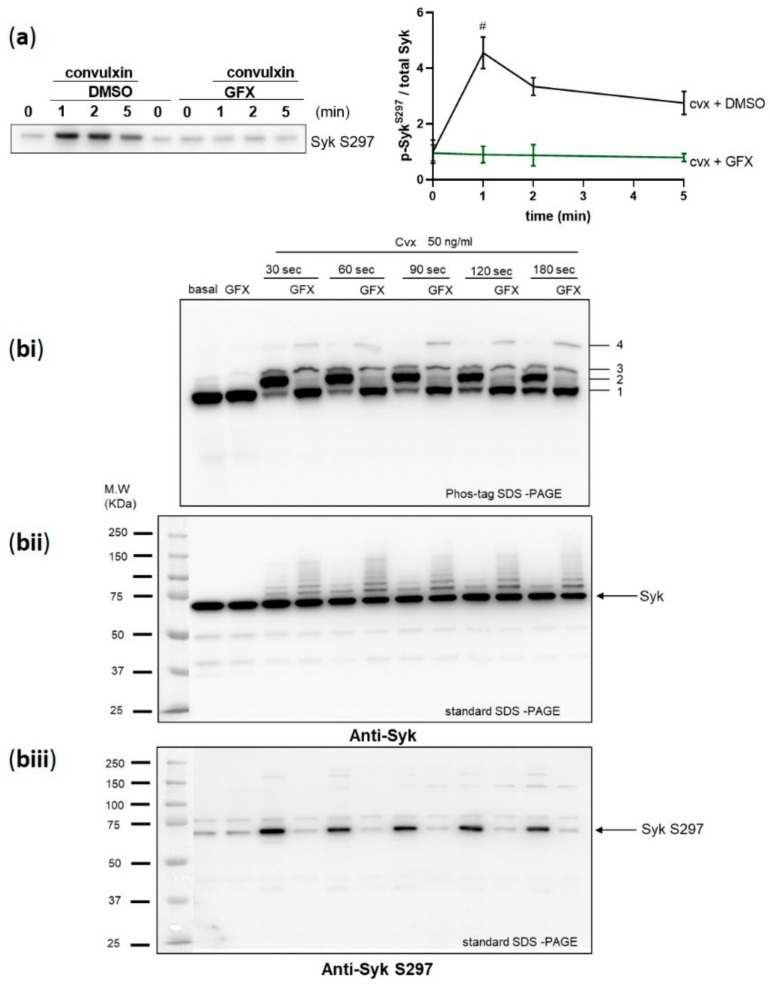
Syk S297 phosphorylation is stoichiometric and completely blocked by PKC inhibition. Washed human platelets were pre-incubated with DMSO as vehicle control or with pan-PKC inhibitor GF109203X (GFX) (5 µM) at 37 °C for 5 min prior to stimulation with 50 ng/mL cvx. (**a**) Platelet aggregation was stopped by adding Laemmli buffer directly in the cuvettes after 1, 2, and 5 min of stimulation under stirring conditions, and Syk S297 phosphorylation was analyzed by standard SDS-PAGE/Western blot analysis compared to total Syk. Quantitative analyses are represented as means ± SD from three independent experiments with platelets from three healthy donors. # *p* < 0.0001, DMSO versus GFX-treated platelets at 1 min. (**bi–biii**) Washed human platelets were stimulated under non-stirring conditions at 37 °C, and platelets were lysed after the indicated time points by using Laemmli buffer for phos-tag SDS-PAGE. (**bi**) Samples were analyzed by phos-tag SDS-PAGE followed by immunoblotting using total Syk antibody. Same samples were analyzed by standard SDS-PAGE followed by immunoblotting using (**bii**) total Syk antibody or (**biii**) anti-Syk S297. Blots are representative of two independent experiments from two healthy donors.

Upon BCR stimulation in murine B-cells, Syk S291, in addition to several tyrosine sites, was a major PKC phosphorylation site in the interdomain-B of Syk which corresponds to S297 in human Syk [24]. Therefore, we tested the role of PKC in Syk S297 regulation in human platelets using a global and potent PKC inhibitor, GF109203X (GFX). Pre-incubation of platelets with GFX completely inhibited cvx-stimulated Syk S297 phosphorylation (Figure 4a).

To analyze quantitative aspects of Syk phosphorylation, we used the phos-tag SDS- polyacrylamide gel-electrophoresis (PAGE) method developed by the group of Koike [31,32]. This method separates phosphorylated and non-phosphorylated forms of proteins including tyrosine kinases depending on their degree of phosphorylation. Immunoblotting using anti-Syk antibody revealed the presence of four migration bands (Figure 4bi). At basal conditions, one major Syk band was detected (band 1), which was not affected by the PKC inhibitor GFX. After 30 sec of activation with cvx, a complete shift of Syk was detected with several bands (2–4), while the major band 1 disappeared and a major band 2 appeared. With GFX pre-treatment, the major band 2 completely disappeared and the basal band 1 reappeared, while the other minor bands (3, 4) were not downregulated. These data indicate that the cvx-induced appearance of band 2 is highly dependent on PKC activity.

In parallel, we analyzed total Syk with the standard SDS-PAGE method and detected only one constant major Syk band in all samples but a row of very minor bands (higher than 72 kDa), especially GFX-treated samples (Figure 4bii), which may be due to Syk ubiquitination [33].

These samples from cvx-treated platelets were also analyzed by standard SDS-PAGE blots using phosphoantibodies. The phosphorylation of Syk S297 was strongly stimulated by cvx and completely inhibited by the PKC inhibitor GFX (Figure 4biii), which closely resembled the appearance/disappearance of band 2 in the phos-tag gel analysis. Overall, the major cvx-induced shift of Syk in phos-tag gels is completely prevented by PKC inhibition and due to Syk S297 phosphorylation.

### 2.5. PKC Activator, PDBu, Stimulates a Stoichiometric Syk S297 Phosphorylation, Which Is Prevented by PKC Inhibition

**Figure 5:**
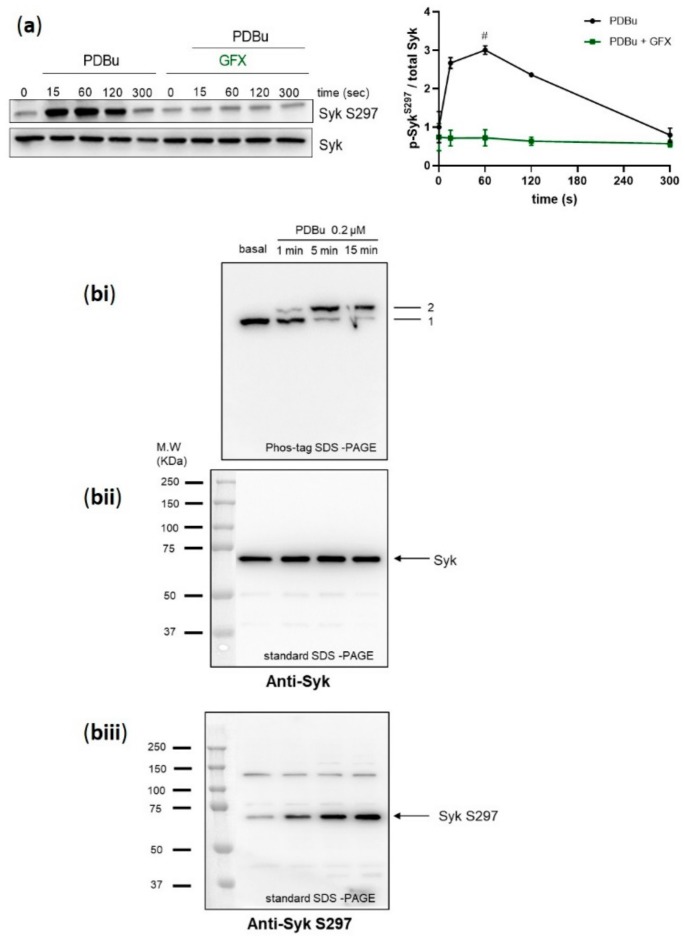
Syk S297 phosphorylation is upregulated by PKC activation in a stoichiometric manner. Washed human platelets were pre-incubated with GF109203X (5 µM) for 5 min at 37 °C prior to stimulation with the phorbol ester PDBu, a global PKC activator (0.2 µM). (**a**) Platelet aggregation was stopped by adding Laemmli buffer directly in the cuvettes after 15, 60, 120, and 300 s of stimulation under stirring conditions, and Syk S297 phosphorylation was analyzed by standard SDS-PAGE/Western blot analysis compared to total Syk. Quantitative analyses are represented as means ± SD from three independent experiments with platelets from three healthy donors. # *p* < 0.0001, untreated versus GFX treated platelets at 1 min. (**bi–biii**) Washed human platelets were stimulated under non-stirring conditions at 37 °C, and platelets were lysed after the indicated time points by using Laemmli buffer for phos-tag SDS-PAGE/immunoblotting analysis. (**bi**) Samples were analyzed by phos-tag SDS-PAGE followed by immunoblotting using total Syk antibody. Same samples were analyzed by standard SDS-PAGE followed by immunoblotting using (**bii**) total Syk antibody or (**biii**) anti-pSyk S297. Blots are representative of two independent experiments from two healthy donors.

To obtain further evidence for PKC-mediated phosphorylation of Syk S297 in human platelets, we tested the effects of a global PKC activator, the phorbol ester phorbol 12, 13-dibutyrate (PDBu). PDBu induced strong platelet aggregation (Supplementary Materials Figure S3) and a very fast and strong phosphorylation of Syk on S297, which appeared within 15 s of stimulation and was stable for the first two minutes (Figure 5a). In addition, we incubated washed human platelets under non-stirring conditions with PDBu and analyzed the Syk phosphorylation profile by phos-tag SDS-PAGE/immunoblotting. At basal conditions, unstimulated Syk showed only one band (band 1), which was completely shifted (band 2) upon PDBu stimulation in a time-dependent manner (Figure 5bi). In parallel, the standard SDS-PAGE/Western blot analysis showed one Syk band for all samples (Figure 5bii) and a time-dependent PDBu stimulated Syk S297 phosphorylation (Figure 5bii,biii). These data support the role of PKC in regulating Syk through S297 phosphorylation.

### 2.6. PDBu-Mediated S297 Phosphorylation Is Independent of Syk and Only Partially Inhibited by PKA

**Figure 6:**
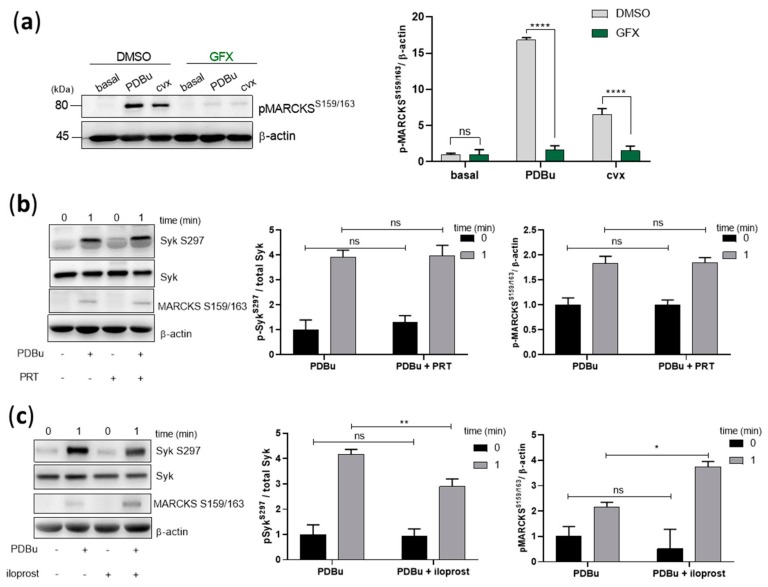
PKC activation induces Syk S297 phosphorylation, which is independent from Syk and only partially inhibited by PKA. Washed human platelets were pre-incubated with DMSO (vehicle control) or with pan-PKC inhibitor, GF109203X (5 µM) at 37 °C for 5 min prior to stimulation with 0.2 µM PDBu or 50 ng/mL cvx. (**a**) Platelet aggregation was stopped by adding Laemmli buffer after 1 min of stimulation under stirring conditions. MARCKS S159/163 phosphorylation was analyzed by standard SDS-PAGE analysis compared to the loading control β-actin. Washed human platelets were pre-incubated with (**b**) Syk inhibitor, PRT-060318 (1 µM) for 5 min or (**c**) iloprost (2 nM) for 3 min prior to stimulation with PDBu. Syk S297 and MARCKS S159/163 phosphorylation was analyzed compared to total Syk or β-actin, respectively. The corresponding quantifications are represented as means ± S.D from three independent experiments with platelets from three healthy donors. **** *p* < 0.0001, ** *p* < 0.01, * *p* < 0.05, ns: not significant.

We then investigated the role of Syk kinase activity on the phosphorylation of S297 mediated by PKC activation. Firstly, we validated the effects of PDBu and GFX by analyzing myristoylated alanine-rich C-*kinase* substrate (MARCKS) phosphorylation, a well-established PKC substrate, also in platelets [34,35]. MARCKS was significantly phosphorylated on S159/163 in response to PDBu and convulxin, which was completely abolished by GFX (Figure 6a). Secondly, Syk inhibition by PRT-060318 did not affect PDBu-induced Syk S297 or MARCKS phosphorylation (Figure 6b), showing that PDBu-caused PKC activation does not require Syk, in contrast to S297 phosphorylation caused by cvx and EB (Figure 3c,d). Thirdly, PDBu/PKC-induced Syk S297 phosphorylation was only partially inhibited by iloprost, and PDBu/PKC-induced MARCKS phosphorylation was even increased by iloprost (Figure 6c). These data suggest that PKC per se was not inhibited by iloprost in intact platelets, but iloprost (PKA) inhibited signaling pathways upstream of PKC activation.

### 2.7. PKC Inhibition Abolished cvx/EB-Induced Syk S297 Phosphorylation But Increased Syk Tyrosine 352 and 525/526 Phosphorylation (Hyperphosphorylation)

**Figure 7:**
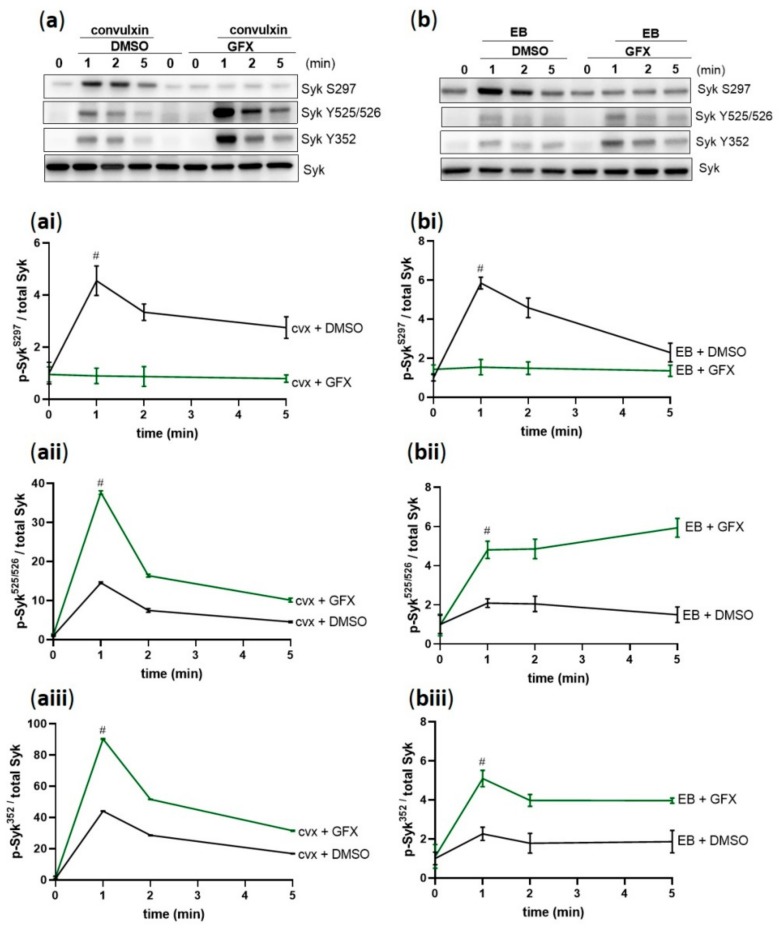
Syk phosphorylation is differentially regulated by PKC. Washed human platelets were pre-incubated with DMSO (vehicle control) or with pan-PKC inhibitor GF109203X (5 µM) at 37 °C for 5 min prior to stimulation with (**a**) 50 ng/mL cvx or (**b**) 0.5% (*v*/*v*) EB. Platelet aggregation was stopped by adding Laemmli buffer after 1, 2, or 5 min of stimulation under stirring conditions. The kinetics of the phosphorylation of Syk on (**ai**,**bi**) S297, (**aii**,**bii**) Y525/526, and (**aiii**,**biii**) Y352 compared to total Syk are represented as means ± SD from three independent experiments with platelets from three healthy donors. # *p* < 0.0001, DMSO-versus GFX-treated platelets at 1 min in response to cvx or EB.

Next, we investigated the role of PKC on Syk activation in GPVI-and GPIbα-mediated platelet activation. PKC inhibition by GFX completely prevented the S297 phosphorylation induced by cvx (Figure 7a,ai) and EB (Figure 7b,bi). In contrast, Syk tyrosine phosphorylation on 525/526 was significantly increased upon stimulation for 1 min compared to the control and subsequently prolonged in both GPVI-(Figure 7a,aii) and GPIbα-mediated signaling (Figure 7b,bii). Syk Y352 phosphorylation showed a similar hyperphosphorylation profile in GPVI-(Figure 7a,aiii), and EB/GPIbα-mediated signaling (Figure 7b,biii). These data demonstrate a striking differential regulation of Syk tyrosine phosphorylation and serine phosphorylation by PKC.

### 2.8. PKC Inhibition Caused Hyperphosphorylation of Y-Sites of Syk Substrates

**Figure 8:**
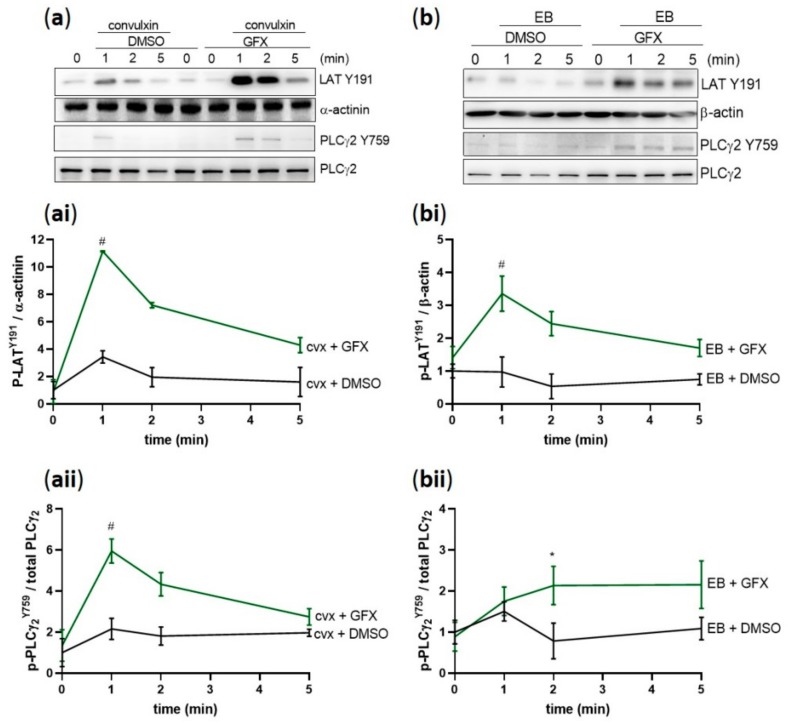
PKC inhibition induces hyperphosphorylation of Y-sites of Syk substrates. Washed human platelets were pre-incubated with DMSO (vehicle control) or with pan-PKC inhibitor GF109203X (5 µM) at 37 °C for 5 min prior to stimulation with (**a**) 50 ng/mL cvx or (**b**) 0.5% (*v*/*v*) EB. Platelet aggregation was stopped by adding Laemmli buffer after 1, 2, or 5 min of stimulation under stirring conditions. The kinetics of the phosphorylation of (**ai**,**bi**) LAT on Y191 and (**aii**,**bii**) PLCγ2 on Y759 compared to the adequate loading control are represented as means ± SD from three independent experiments with platelets from three healthy donors. # *p* < 0.0001, * *p* < 0.05, DMSO-versus GFX-treated platelets at 1 min or 2 min in response to cvx or EB, respectively.

To validate the possible functional consequence of Syk tyrosine hyperphosphorylation, we investigated the phosphorylation of Syk downstream substrates linker adaptor for T cells (LAT) and PLCγ2 on Y191 and Y759, respectively. Cvx-and EB-induced LAT phosphorylation was remarkably increased in the presence of the PKC inhibitor compared to the control (Figure 8a,b,ai,bi). In addition, PLCγ2 also showed an increase and prolongation in the Y759 phosphorylation in both signaling pathways cvx/GPVI (Figure 8a,aii) and EB/GPIbα (Figure 8b,bii). These data demonstrate that Syk hyperphosphorylation is associated with direct increased tyrosine phosphorylation of LAT and PLCγ2.

### 2.9. PKC Inhibition Enhances cvx/EB-Mediated InsP1 Production and Intracellular Ca^2+^ Mobilization

**Figure 9:**
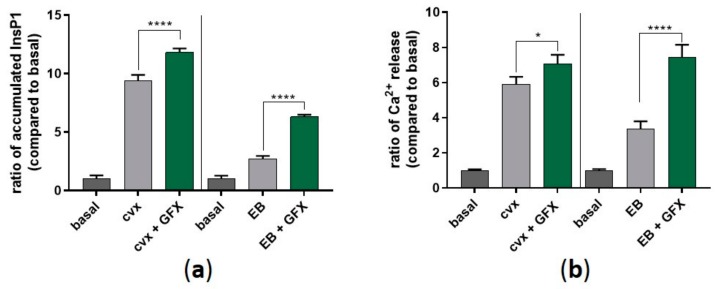
PKC inhibition significantly enhances InsP1 accumulation and intracellular Ca^2+^ mobilization. (**a**) Washed human platelets were pre-treated with GF109203X (5 µM) as previously described in the presence of 1 mM LiCl. Platelet aggregation was stopped after 5 min using lysis buffer provided by the manufacturer. (**b**) Washed human platelets were pre-incubated with Fluo-3 AM (5 µM) for 30 min at 37 °C, and platelet stimulation was performed directly before measurement. Ca^2+^_i_ mobilization was monitored for 120 s by flow cytometry. Quantitative data are represented as means ± SD from three independent experiments with platelets from three healthy donors. **** *p* < 0.0001, * *p* < 0.05.

Furthermore, we aimed to measure the activity of PLCγ2 by monitoring intracellular inositol triphosphate (InsP3) production (via inositol monophosphate (InsP1) accumulation) and intracellular Ca^2+^ mobilization. In the presence of GFX, cvx-and EB-evoked InsP3 production was significantly enhanced compared to the control in the absence of the PKC inhibitor (Figure 9a). Moreover, this InsP3 production, mediated by cvx and EB, led to a significant increase of intracellular Ca^2+^ mobilization, which was even more pronounced in the presence of GFX (Figure 9b). These data show that Syk hyperphosphorylation is followed by an increase in LAT/PLCγ2 phosphorylation leading to an increase of InsP3 production and subsequent Ca^2+^ mobilization.

## 3. Discussion

In this study, we show (1) that activation of human platelets via GPVI and GPIbα, as well as by pharmacological activation of PKC, caused phosphorylation of Syk S297, and (2) that pharmacological PKC inhibition or receptor-linked inhibition of PKC by cAMP/PKA abolished Syk S297 phosphorylation, but enhanced Syk tyrosine phosphorylation/activity and phosphorylation of Syk substrates. These data suggest that Syk S297 phosphorylation is mediated by PKC and represents a mechanism of feedback inhibition of Syk in human platelets.

Several lines of evidence show that a classical, diacylgycerol/phorbolester-responsive protein kinase C (PKC) phosphorylates Syk S297 in human platelets:

Increased Syk S297 phosphorylation is observed when ADP receptors, GPVI, and GPIbα are stimulated by selective agonists, which also stimulate PLC and PKC activation.

- GPVI-and GPIbα-caused Syk S297 phosphorylation is Syk-dependent and requires, at least predominantly, the release of secondary mediators ADP and TxA2.
- GPVI-/GPIbα-induced Syk S297 phosphorylation is blocked by a selective PKC inhibitor (GFX) and mimicked by an established, membrane permeable PKC activator (PDBu).
- This agonist-induced Syk S297 phosphorylation is also inhibited by the cAMP/PKA pathway, which is known to strongly inhibit receptor-stimulated Ca2^+^ responses and PKC activation [30,36,37].
- The peptide sequence around Syk S297* 9ISRIKSYS*FPKPGHR) in humans (in the murine form S291, ISRIKSYS*FPKPGHK) agrees well with the PKC phosphosite consensus sequence [24] (see also other phosphosite databases).

While the evidence for a conventional PKC as a responsible Syk S297 kinase in intact human platelets is quite strong, we do not know which PKC isoform (or perhaps more than one) is involved. PKC isoforms consist of conventional forms (α, βI, βII, γ) activated by Ca^2+^/diacylglycerol, novel forms (δ, ε, η, θ) activated by diacylglycerol alone, and atypical forms, which are diacylglycerol-independent. Human and mouse platelets highly express the conventional PKC isoforms α/β and the novel isoforms δ and θ [36,38], which all have important but diverse roles in platelets, which may be inhibitory or activating [36,39]. Therefore, we cannot rule out that more than one member of the PKC family is capable of phosphorylating platelet Syk S297. Previously, a functional interaction of PKCα with both Src and Syk was reported for human platelets, which occurred together with a Syk-dependent tyrosine phosphorylation of PKCα, but not the other way around, i.e., PKCα-dependent serine phosphorylation of Syk [40]. An Src/SFK-dependent phosphorylation and activation of PKCδ was observed in platelets, as well as a PKCδ-dependent activation of Syk in endothelial cells [41], indicating interactions between Syk, other tyrosine kinases, and the PKC family.

In our study, we observed a rapid receptor-mediated Syk S297 phosphorylation (onset within 15 s of stimulation), which reached stoichiometry and then declined back to the basal status. This conclusion was possible due to the combination of regular Western blot/pSyk S297 phosphoantibody analysis with phos-tag analysis. The initial major Syk band in phos-tag gels was almost completely shifted to another major band, which was completely shifted back to the basal state when platelets where pre-treated with the PKC inhibitor GFX. This major band was the only Syk band with this property, a pattern also observed by regular Syk pS297 phosphoantibody analysis. The conclusion that these effects are PKC-mediated is strongly supported by observations made with the direct PKC activator PDBu. Treatment of platelets with PDBu caused, as detected in phos-tag gels, a time-dependent, complete shift of this major Syk band, which was also completely prevented by the PKC inhibitor. Compared to cvx treatment, no minor additional shifted band appeared in the PDBu treatment detected in phos-tag gels. The phos-tag data with PDBu additionally suggest that PKC phosphorylates only one major site (S297) in Syk, although Syk contains a number of serine/threonine protein kinase phosphosites [23]. The other, minor cvx-shifted Syk bands, detected in phos-tags, are not downregulated by GFX and correspond to tyrosine-phosphorylated sites of Syk. Altogether, Syk S297 phosphorylation in human platelets is mediated by PKC, in a stoichiometric and reversible manner.

While the phosphorylation of Syk S297 can reach stoichiometry in our studies with human platelets, the corresponding stoichiometry of Syk tyrosine phosphorylation appears to be much smaller. Interestingly, Syk phosphorylation data obtained with murine B-cells suggested that phosphorylation of Syk at tyrosines is restricted to a subset of the kinase physically associated with ITAM-containing membrane proteins, whereas the interdomain serine sites are also phosphorylated in Syk, when Syk is not membrane-bound [24].

Another feature of GPVI/GPIbα-mediated Syk phosphorylation and activation in platelets is the transient nature of both tyrosine (Y352, Y525/Y526) and serine (S297) phosphorylation. Onset and decline of Cvx/EB stimulated SykS297 phosphorylation resemble the time course of Syk Y352/Y525/526 phosphorylation, which indicates Syk activation [18,19]. The state of protein phosphorylation is determined, for each individual site, by both protein kinases and protein phosphatases, and it can be decreased, for example, by kinase inhibition, phosphatase activation, or both. When murine B-cell Syk activation is discontinued, both Syk and its substrates are rapidly tyrosine-dephosphorylated, and the downstream signaling effects end [22,42]. Several protein tyrosine phosphatases were identified to dephosphorylate tyrosine phosphorylated Syk including the SH2 domain-containing tyrosine protein phosphatase-1 (SH-1; PTPN6), e.g., T-cell ubiquitin ligand 2 (TULA-2; UBASH3B) [22], and most of them are present in human platelets [29,38]. TULA-2 is an unconventional protein tyrosine phosphatase with a ubiquitin-associated domain (UAB), a SH3 domain and a special histidine phosphatase domain, and TULA-2 binds and uses Syk as substrate [43,44]. This is of functional significances since the state of Syk tyrosine phosphorylation is reduced in cells overexpressing TULA-2, but enhanced when TULA-2 is impaired or deficient. In both murine and human platelets, strong evidence was obtained that TULA-2 suppresses the GPVI-induced, FcRγ-mediated Syk activation by dephosphorylating primarily Syk Y346 (Y352 in human) [45], which also belongs to the interdomain-B. Compared to the tyrosine dephosphorylation of Syk, there is no information available on serine/threonine dephosphorylation, which is most likely catalyzed by one or more of the many serine/threonine protein phosphatases present in human platelets [29].

A remarkable result reported here is that the specific inhibition of Syk S297 phosphorylation by the PKC inhibitor GFX closely coincided with enhanced cvx/EB-stimulated Syk Y352/Y525/526 phosphorylation (indicating enhanced Syk activity), enhanced Syk substrate phosphorylation (LAT, PLCγ2), and enhanced cvx/EB-induced InsP1/InsP3 increase. Considering the dynamic aspects of Syk activity by phosphorylation/dephosphorylation observed in other cells [22], the effects observed here are substantial. Interestingly, the PKC inhibitor GFX was reported to enhance GPVI-mediated Syk phosphorylation at Y525/526 without affecting the Y352 and Y323 sites in human platelets, effects not observed with murine platelets [46]. However, direct PKC substrates were not investigated. In our studies, platelet agonists, but also the PKC activator PDBu, stimulated not only Syk S297 phosphorylation, but also, used here as a control, the phosphorylation of MARCKS, a well-established PKC substrate [35], and both responses were prevented by the PKC inhibitor GFX.

There is another line of evidence for the conclusion that PKC inhibition enhances Syk and Syk substrate tyrosine phosphorylation with the possible involvement of Syk S297 phosphorylation. We recently showed that both cAMP- and cGMP-elevating platelet inhibitors (iloprost, riociguat), enhance Syk tyrosine phosphorylation/Syk activity [28]. Now, we show that iloprost (PKA pathway) strongly inhibits cvx/EB-stimulated Syk S297 phosphorylation. It is likely that, under our conditions, iloprost inhibits the platelet effects of ADP and TxA2, which, when released during cvx/EB stimulation, are important secondary messengers and (as we clearly showed for ADP) are able to stimulate PKC-mediated Syk S297 phosphorylation.

**Figure 10:**
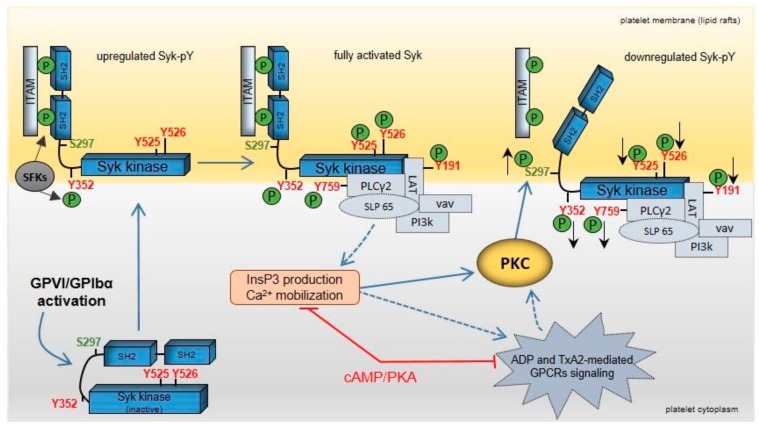
Model showing feedback regulation of Syk upon GPVI and GPIbα activation in human platelets. Clustering of GPVI and GPIbα upon agonist binding activates platelets initiated by src family kinases (SFKs) and the spleen tyrosine kinase (Syk). SFK activation leads to dual phosphorylation of the “immunoreceptor tyrosine-based activation motifs” (ITAMs), which represents a docking site for the two src homology 2 (SH2) domains of Syk. The interaction between Syk-(SH2)^2^ domains and dually tyrosine-phosphorylated ITAMs recruits and translocates Syk to lipid rafts of the platelet cell membrane. Syk tyrosine phosphorylation of Y525/526 (kinase domain) and Y352 (interdomain-B) by autophosphorylation and SFKs leads to a fully activated kinase. Consequently, specific Syk substrates and regulatory proteins, such as LAT, PLCγ2, SLP65, vav, and phosphoinositide-3 kinase (PI3k) are tyrosine-phosphorylated and recruited to a “LAT signalosome” complex. Activation of the Syk substrate PLCγ2 produces the release of ADP (from platelet δ-granules) and of TxA2 via elevation of InsP3 and Ca^2+^. These secondary messengers enhance platelet activation via specific GPCRs, which also involves activation of distinct PKC isoforms. GPVI/GPIbα not only induce Syk tyrosine phosphorylation but also Syk phosphorylation on S297, which is located within the interdomain-B. GPVI/GPIbα-induced Syk S297 phosphorylation is mediated by PKC and decreases Syk tyrosine phosphorylation/activation. The cAMP/PKA pathway reduces the PKC response. It is proposed that Syk S297 phosphorylation represents an important mechanism for Syk feedback inhibition.

Our study demonstrates the regulation of Syk S297 phosphorylation in a primary human cell, the platelet, in response to several agonists such as ADP, cvx, and EB (Figure 10). Increased S297 phosphorylation is mediated by PKC and negatively affects the tyrosine phosphorylation state of Syk at both the interdomain-B (Y352) and the kinase domain (Y525/526). The S297 serine site of Syk is located at a crucial site of Syk regulation and activation, the interdomain-B, just at the border of the c-terminal SH2-domain [18,20] (Figure 10).

ITAM-and/or tyrosine phosphorylation-dependent Syk activation occurs at the membrane and alters the interaction of the various Syk domains, releasing the autoinhibition and producing an active kinase. Although there is functional and structural information that the interdomain-B with its phosphorylation and various interacting proteins is essential for the transition of inactive to activated Syk, the exact molecular mechanism(s) for this transition inside a cell are unknown. A major part of the crucial interdomain-B (amino acids 262–337) was unfortunately not defined in the otherwise impressive crystal structure of full-length inactive Syk [20], perhaps due to the flexible nature of interdomain-B.

The role of Syk phosphorylation including serine 297 (S291 in murine Syk) was extensively investigated by two groups in the chicken B-cell model system DT40, but discrepant functional data were obtained [23,24]. With murine Syk, PKC-mediated Syk S291 phosphorylation enhanced the ability of Syk to couple and activate the antigen receptor complex, perhaps involving the interaction of the interlinker domain B with the chaperone prohibitin [24]. These murine Syk effects were impaired when the murine S291A Syk mutant was tested. The other investigation with human Syk reported the presence of multiple Syk phosphorylation sites and also multiple Syk-interacting proteins. The major Syk phosphosite S297 (when phosphorylated) was reported to recruit the adapter protein 14-3-3γ, which prevented the translocation of Syk to the plasma membrane and subsequent Syk activation and downstream effects [23]. These effects were impaired when the experiments were carried out with the human Syk S297A mutant. The discrepancies between these two important studies are yet to be resolved, but a striking difference was the interaction with adapter proteins recruited to the phosphorylated interdomain-B.

Enhanced Syk tyrosine phosphorylation mediated by downregulation of Syk S297 phosphorylation through PKC inhibition is very likely due to decreased tyrosine dephosphorylation. There are several possible mechanisms via which Syk S297 phosphorylation could regulate this.

(a) Syk S297 phosphorylation could alter the structure/properties of Syk, which could make Syk a better substrate for tyrosine phosphatases (e.g., TULA-2).
(b) Syk S297 phosphorylation could recruit adapter proteins to this site (e.g., 14-4-3γ, prohibitin), which could decrease Syk translocation to ITAM proteins and, therefore, diminish Syk tyrosine phosphorylation/activation.
(c) PKC could phosphorylate and activate TULA-2/other tyrosine phosphatases resulting in enhanced Syk tyrosine dephosphorylation. However, there is no evidence for PKC-mediated phosphorylation and activation of TULA-2 [43].
(d) PKC could regulate other proteins, which affect Syk phosphorylation/dephosphorylation.

It is also possible that there are cell-specific mechanisms of Syk regulation, perhaps due to different binding/adapter proteins. These aspects should be investigated with platelets in the future. Our data with human platelets suggest that ITAM-dependent activation of Syk tyrosine phosphorylation is limited by Syk-dependent, PKC-mediated increased S297 phosphorylation, which can be prevented by PKC inhibition. This may represent a feedback inhibition important to prevent hyperactivation of Syk. Feedback inhibition is often observed with receptor-dependent signaling including tyrosine kinases. For example, PKC-stimulated phosphorylation of the epidermal growth factor receptor (EGFR) was reported to represent negative feedback inhibition of this signaling by converting the active EGFR dimer back to the inactive monomer [47]. Of special interest with respect to soluble tyrosine kinases, it was reported that PKCβ-mediated phosphorylation of the Bruton-tyrosine kinase BTK on S180 prevented membrane association and further tyrosine phosphorylation/activation of BTK in B and mast cells [48].

Considering the central role of Syk in immune cells and platelets, its established role in many disease processes including myeloid malignancies [15,49,50], and its increasingly recognized role as a therapeutic target [51,52], it will be now very important to elucidate the precise mechanism via which Syk is controlled by S297 phosphorylation.

Clinically used Syk inhibitors not only target Syk but also other tyrosine protein kinases. A clear direction of further development would be to find more specific Syk inhibitors, perhaps inhibitors, which do not directly target the kinase domain but a Syk-specific regulatory/allosteric domain such as the interdomain-B. Another problem with clinically used protein kinase inhibitors and also Syk inhibitors is the emergence of resistance, which was very recently addressed in a large genomic screen in acute myloid leukemia (AML) [50]. This screen identified activation of the RAS/MAPK/ERK pathway as one major mechanism of resistance to Syk inhibitors, which was further validated in AML cell lines with innate and acquired resistance to Syk inhibitors. The authors discussed their data, whereby resistance to tyrosine kinase inhibitors during targeted therapies may be due to secondary mutations of the target kinase, activation of other upstream stimulators or downstream effectors of the pathway, or the activation of parallel pathways. These very recent clinical findings highlight that improvement of our understanding of Syk regulation at the tyrosine kinase level directly, but also at the level of both upstream and downstream of this crucial component of platelet, immune, and myeloid cell activation, is essential for further advances in the area of Syk pathophysiology and inhibitors. Our present study on the feedback inhibition of Syk is perhaps one small step in this direction and also provides a human-relevant model of Syk signaling.

## 4. Materials and Methods

### 4.1. Ethics Approval

All healthy donors gave their informed consent for inclusion before they participated in the study. The study was conducted in accordance with the Declaration of Helsinki, and the protocol was approved by the local Ethics Committee of the University Medical Center Mainz (Study No. 837.302.12; 25.07.12; FF109/2015).

### 4.2. Preparation and Stimulation of Human Platelets

Whole blood was collected in citrate tubes from healthy volunteers who did not take any medication at least 10 days before the experiments. Platelet washing and the isolation procedure were performed as previously described [28]. A modified washing procedure was used for the experiments with ADP stimulation. PRP was diluted 1:1 with CGS buffer (120 mM NaCl, 12.9 mM Tri-Na-citrate, 30 mM glucose, pH 6.5) then centrifuged at 400× *g* for 10 min at room temperature (RT). The platelet pellet was resuspended directly with HEPES buffer (150 mM NaCl, 5 mM KCl, 1 mM MgCl_2_, 10 mM glucose, 10 mM HEPES, pH 7.4). Human washed platelets were adjusted to 3 × 10^8^ platelets/mL and kept at 37 °C prior to stimulation under stirring conditions with the corresponding platelet agonist or activator in the presence or absence of vehicle control or effectors.

### 4.3. Platelet Aggregometry

Aggregation of washed human platelets was monitored by light transmission aggregometry (LTA) using an Apact4S Plus aggregometer (DiaSys Greiner, Flacht, Germany). Platelets were stimulated by ADP (Sigma-Aldrich, Saint Louis, MO, USA), convulxin (Enzo life sciences, Lausen, Switzerland), echicetin beads (EB) (prepared as previously described [28,53]), or PDBu (Sigma-Aldrich, Saint Louis, MO, USA) under stirring conditions (1000 s^−1^) at 37 °C. Platelets were pre-treated with different effectors prior to stimulation: Src family kinase inhibitor PP2 (Abcam, Cambridge, UK), Syk inhibitors OXSI-2 (Merck, Darmstadt, Germany) and PRT-060318 (Sellckem, Houston, TX, USA), PKC pan-inhibitor GF109203X (Enzo life sciences, Lausen, Switzerland), or cAMP-elevating agent iloprost (Bayer AG, Leverkusen, Germany).

### 4.4. SDS-PAGE and Western Blot Analysis

Platelet samples for Western blot analysis were prepared by adding 3× Lämmli buffer (200 mM tris/HCl, 15% (*v*/*v*) glycerol, 6% (*w*/*v*) SDS, 0.06% (*w*/*v*) bromphenol blue, 1:10 β-mercaptoethanol) directly in the aggregation cuvettes to stop platelet responses, then boiled at 95 °C for 10 min under gentle shaking. Platelet proteins were separated by electrophoresis using 8% SDS-polyacrylamide gels followed by immunoblotting as previously described [28].

Phospho-antibodies against Syk S297, Syk Y525/526, Syk Y352, LAT Y191, PLCγ2 Y759, and MARCKS S159/163 (Cell Signaling Technologies, Danvers, MA, USA) were used diluted 1:1000 in 5% BSA or 1:700 for MARCKS. Blots probed with phospho-antibodies were stripped and reprobed with a corresponding antibody detecting the total protein, anti-Syk, or anti-PLCγ2 (Santa Cruz Biotechnology, Santa Cruz, CA, USA), or other loading controls anti-β-actin or anti-α-actinin (Cell Signaling Technologies, Danvers, MA, USA) diluted 1:1000 in 5% BSA. After incubation with the appropriate secondary antibody, horseradish peroxidase (HRP)-conjugated goat anti-rabbit and anti-mouse IgG (BioRad Laboratories, Hercules, CA, USA) enhanced chemiluminescence (ECL) detection was performed using Fusion FX7 (Vilber Loumat GmbH, Eberhardzell, Germany).

### 4.5. Zn^2+^-Phos-Tag^tm^-SDS-PAGE and Western Blot Analysis

The phos-tag method was developed and described by the group of Koike [31,32] and used here for platelet Syk. Platelets were lysed using 3× Laemmli buffer for phos-tag (200 mM tris, 15% (*v*/*v*) glycerol, 6% (*w*/*v*) SDS, 2% (*w*/*v*) bromphenol blue, 1:10 β-mercaptoethanol) in the absence of EDTA. Samples were boiled at 95 °C for 10 min under gentle shaking. Platelet proteins were separated by electrophoresis using gels containing 6% *w/v* polyacrylamide, 35 µM phos-tag^TM^ compound, and 69 µM ZnCl_2_ in the separating gel. Then, 1× phos-tag running buffer was used for gel electrophoresis (0.1 M tris, 0.1 M MOPS, 0.1% (*w*/*v*) SDS, 5 mM sodium bisulfite, pH 7.8). Prior to protein membrane transfer, gels were washed twice for 10 min with 1× transfer buffer containing 1 mM EDTA to remove the Zn^2+^ ions. A third washing step was performed with 1× transfer buffer without EDTA before the proteins were transferred to polyvinylidene difluoride (PVDF) membranes using a transfer buffer for phos-tag (25 mM tris, 192 mM glycine, 10% (*v*/*v*) methanol, 5% (*w*/*v*) SDS, pH 8.4).

Immunoblotting was performed as previously described [28]. Mouse monoclonal Syk antibody (Santa Cruz Biotechnology, Santa Cruz, CA, USA) was used as primary antibody (1:1000 in 5% BSA) and HRP-conjugated mouse antibody was used as secondary antibody (1:5000 in 5% BSA).

### 4.6. Inositol Monophosphate (InsP1) Measurement

Accumulated inositol monophosphate (InsP1), reflecting the produced inositol triphosphate (InsP3) upon platelet stimulation, was measured by using the IP-One ELISA kit (Cisbio, Codolet, France) in the presence of LiCl (1 mM), which prevents the degradation of InsP1 into myo-inositol. Washed platelets (3 × 10^8^ platelets/mL) were stimulated with convulxin or EB in the absence or presence of effectors at 37 °C under stirring conditions, lysed after 5 min of activation, and centrifuged at 16,000× *g* for 10 min at 4 °C. InsP1–HRP conjugate and anti-InsP1 monoclonal antibody were pre-incubated with platelet lysates for 3 h prior to measurement, and InsP1 was quantified according to the manufacturer’s instructions.

### 4.7. Intracellular Ca^2+^-Release Measurement

Washed platelets (3 × 10^8^ platelets/mL) were loaded for 30 min at 37 °C with 5 µM Fluo-3 acetoxymethyl (AM) esters (Life Technologies, Carlsbad, CA, USA), a Ca^2+^ indicator dye with excitation/emission of 506 nm/526 nm for the Ca^2+^-bound form. Intracellular Ca^2+^ was monitored as mean fluorescence intensity for 2 min detected by flow cytometry (BD FACSCANTO II, BD Biosciences, Heidelberg, Germany) after stimulation with cvx or EB, without supplementation of extracellular Ca^2+^ and in the absence or presence of effectors. Data were analyzed by FACSDiva software v6.1.3 (BD Biosciences, Heidelberg, Germany) expressing the ratio of mean fluorescence intensity over time of treated platelets vs. basal platelet condition.

### 4.8. Statistical Analysis

Each dataset represents at least three different experiments from at least three different healthy volunteers when data are expressed as means ± standard deviation (SD). Statistical analysis was performed using GraphPad Prism 8 (GraphPad Software, San Diego, CA, USA). One-way or two-way ANOVA, followed by Tukey’s multiple comparison test for comparison of more than two groups, or the two-tailed Student’s *t*-test for comparison of two groups were used. A *p*-value < 0.05 was considered as significant.

## Supplementary Materials

Supplementary Materials can be found at https://www.mdpi.com/1422-0067/21/1/176/s1. Supplementary Figures 1–3/File1.

## Abbreviations

ACK1	Activated CDC42 kinase 1
ADP	Adenosine diphosphate
BCR	B-cell antigen receptor
BTK	Bruton-tyrosine kinase
cAMP	Cyclic adenosine monophosphate
cGMP	Cyclic guanosine monophosphate
cvx	Convulxin
EB	Echicetin beads
GFX	2-[1-(3-Dimethylaminopropyl)indol-3-yl]-3-(indol-3-yl) maleimide
HRP	Horseradish peroxidase
InsP1	Inositol monophosphate
InsP3	Inositol triphosphate
ITAM	Immunoreceptor tyrosine-based activation motif
JAK	Janus kinase
LAT	Linker adaptor for T cells
MARCKS	Myristoylated alanine-rich C- kinase substrate
PDBu	Phorbol 12, 13-dibutyrate
PI3k	Phosphoinositide- 3 kinase
PKA	Protein kinase A
PKC	Protein kinase C
PLCγ2	Phospholipase Cγ2
S	Serine
PAGE	Polyacrylamide gel-electrophoresis
SH2	Src homology 2
Syk	Spleen tyrosine kinase
T	Threonine
TxA2	Thromboxane A2
vWF	Von Willebrand factor
Y	Tyrosine
