# Supplementary material for: Feedback Regulation of Syk by Protein Kinase C in Human Platelets
Source: Int J Mol Sci. 2019 Dec 25;21(1):176. doi: 10.3390/ijms21010176 (PMC6981976; doi:10.3390/ijms21010176)
Supplement: Supplementary file 1 [file ijms-21-00176-s001.zip › Supplementary-figures-file-1.docx]

**Supplementary Figures**

**
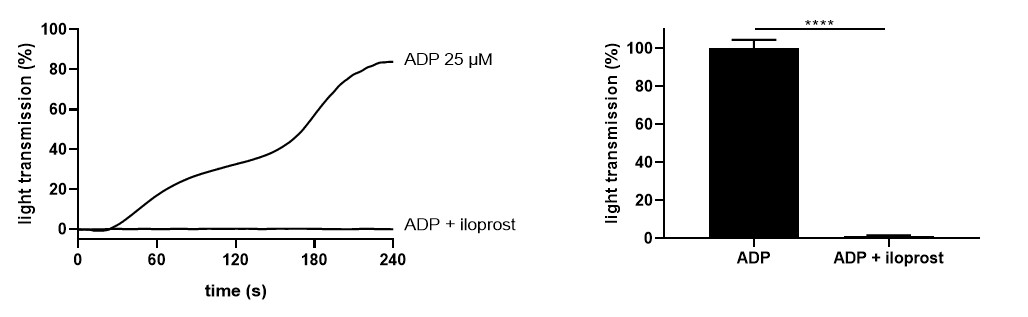
**

**Figure S1.** Iloprost abolishes ADP-induced platelet aggregation**.** Washed human platelets were pre-incubated with iloprost (2 nM; 3 min at 37°C) prior to stimulation with 25 µM ADP. Quantitative data are represented as mean ± S.D from 3 independent experiments with platelets from 3 healthy donors. ****p<0.0001


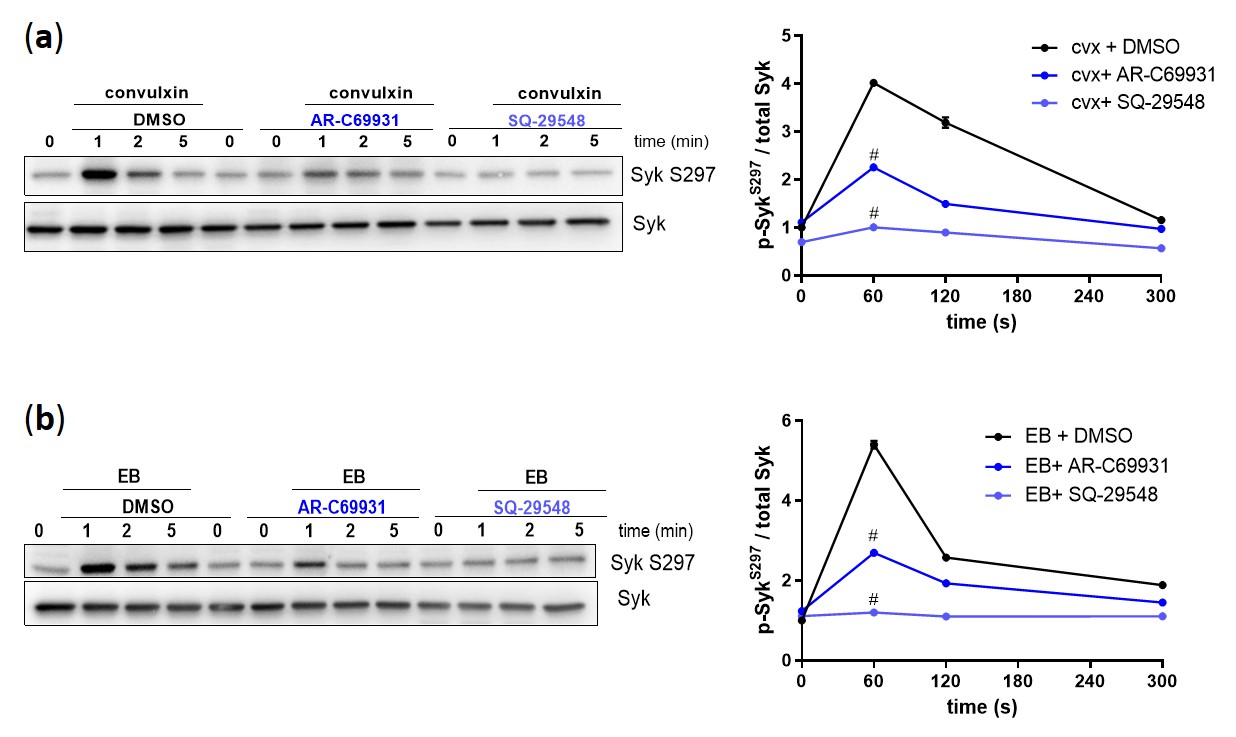


**Figure S2.** Cvx- and EB-induced S297 phosphorylation is significantly dependent on the secondary mediators ADP and TxA2. Washed platelets were pre-incubated for 5 min at 37°C with vehicle control (DMSO) or with the P2Y12 inhibitor AR-C69931 (0.1 µM), or with the TxA2 receptor antagonist SQ-295448 (1 µM) prior to stimulation with (a) 50 ng/ml convulxin or (b) EB, under stirring conditions. Platelet aggregation was stopped after 1, 2 and 5 min by adding directly Laemmli buffer. Samples were boiled at 95°C for 10 min. Immunoblotting using antibody against Syk S297 was performed. The corresponding quantification of Syk S297 phosphorylation compared to total Syk are represented as mean ± S.D from 3 independent experiments with platelets from 3 healthy donors. #p<0.0001, DMSO versus inhibitor (AR-C or SQ) treated platelets at 1 min after stimulation at 37°C under stirring conditions.


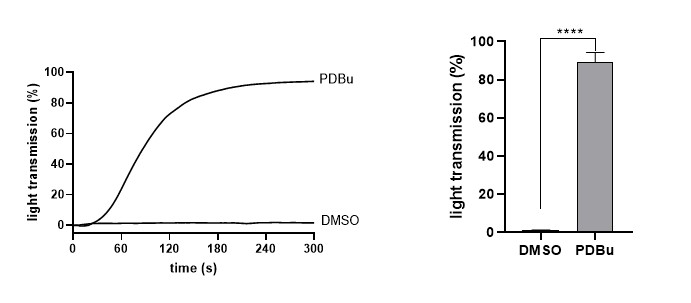


**Figure S3.** The PKC activator, PDBu, induces a strong and fast platelet aggregation. Washed platelets were stimulation with PDBu (0.2 µM) or with vehicle control, DMSO (0.002 %). Quantitative data are represented as mean ± S.D from 3 independent experiments with platelets from 3 healthy donors. ****p<0.0001
